# Mechanisms of skeletal muscle atrophy in type 2 diabetes mellitus

**DOI:** 10.3389/fphys.2025.1607873

**Published:** 2025-06-25

**Authors:** Jingyi Yang, Yingdong Wang, Yuzhe Xu, Xinqi Jia, Fangping Lu

**Affiliations:** ^1^ Department of Pathophysiology, Mudanjiang Medical University, Mudanjiang, China; ^2^ Department of General Surgery, Mudanjiang Medical University Affiliated Hongqi Hospital, Mudanjiang, China

**Keywords:** type 2 diabetes mellitus, endoplasmic reticulum stress, apoptosis, skeletal muscle atrophy, proteostasis

## Abstract

**Introduction:**

ERS-induced apoptosis may play a pivotal role in diabetic skeletal muscle atrophy. However, the specific mechanisms by which ERS regulates skeletal muscle atrophy in diabetes remain unclear. The research examines the impact of endoplasmic reticulum stress (ERS) on skeletal muscle atrophy in type 2 diabetes mellitus (T2DM) mice.

**Methods:**

Leptin receptor-deficient *Db/db* mice (n = 7, 24-week-old, male) were employed as a type 2 diabetes model, while age-matched male C57BL/6J mice (n = 7) served as normal controls. Pathway enrichment analysis of differentially expressed genes was performed based on transcriptome sequencing data, focusing on apoptosis, ERS, and ubiquitin-proteasome pathways. Skeletal muscle morphology was assessed via anatomical observation, Laminin Staining, and immunoblotting analysis (WB). WB was used to detect ERS markers (ATF6, p-eIF2α, Bip, p-JNK, Chop), apoptosis-related proteins (Bcl2, Bax, Cleaved Caspase-3, CytC), p-Akt, and muscle atrophy marker Atrogin1.

**Results:**

Transcriptomic enrichment analysis confirmed specific activation of apoptosis, ERS, and ubiquitin-proteasome pathways. WB revealed upregulated ERS-related proteins, increased apoptotic proteins, decreased p-Akt expression, elevated Atrogin1 levels, and enhanced proteolytic activity. *Db/db* mice exhibited significant skeletal muscle atrophy, with Laminin Staining demonstrating reduced cross-sectional area (CSA) of muscle fibers.

**Discussion:**

These findings uncovers a dual regulatory mechanism underlying diabetic muscle atrophy. The diabetic skeletal muscle microenvironment exhibits elevated oxidative stress and significantly enhanced ER stress, which promotes direct muscle atrophy through ER stress sensor-mediated apoptosis. Concurrently, sustained ER stress suppresses Akt activity while upregulating the muscle-specific E3 ubiquitin ligase Atrogin1, thereby accelerating proteolysis and inducing indirect muscle wasting. These findings provide crucial mechanistic insights into diabetic skeletal myopathy, highlighting the ER stress signaling network as a promising therapeutic target for mitigating muscle atrophy in diabetes.

## 1 Introduction

Diabetes mellitus (DM) represents a global metabolic dysregulation syndrome, where T2DM is pathophysiologically characterized by insulin resistance. T2DM is often accompanied by various complications, including skeletal muscle atrophy. Skeletal muscle, serving as the principal regulator of systemic glucose homeostasis, functions as the primary insulin-target tissue that mediates postprandial glucose disposal through GLUT4-dependent uptake mechanisms (Sylow et al., 2021). Skeletal muscle atrophy is pathologically associated with diverse clinical contexts spanning: (1) age-related sarcopenia; (2) chronic diseases; and (3) malignancy-associated cachexia (Ishii et al., 2008; Mendes et al., 2015; Zhang H. et al., 2023; Zhang et al., 2024). Emerging evidence demonstrates that dysregulated mROS production and caspase-3 activation constitute pathophysiological mechanisms driving proteolytic degradation in atrophying muscle (Plant et al., 2009; Min et al., 2011; Zhang et al., 2024).

The endoplasmic reticulum (ER) serves as a central organelle for cellular proteostasis, coordinating protein synthesis, post-translational modification, secretory trafficking, and folding quality control (Yoshida, 2007). ERS can be triggered by various pathological and physiological factors, including Ca2+ dysregulation, redox imbalance, defective protein folding, and other stressors (Jiang et al., 2021). In patients with T2DM, obesity is frequently associated with enhanced adipokine secretion, which subsequently promotes excessive reactive oxygen species (ROS) generation and induces systemic oxidative stress (Fernandez-Sanchez et al., 2011). As the primary defense mechanism against oxidative damage, the endogenous antioxidant system mobilizes crucial enzymes including superoxide dismutase (SOD) and catalase (CAT) to establish the first-line protective barrier against oxidative assault (Jomova et al., 2024). This compensatory upregulation represents a critical adaptive response to maintain redox homeostasis. Furthermore, the persistent oxidative imbalance contributes to the accumulation of misfolded proteins, thereby triggering endoplasmic reticulum (ER) stress through impaired protein folding capacity (Ong and Logue, 2023). Physiological Unfolded Protein Response (UPR) activation under moderate ERS orchestrates proteostatic adaptation through three transmembrane sensors. Conversely, sustained ERS drives terminal UPR signaling that activates caspase-dependent apoptotic cascades, culminating in programmed cell death (Park et al., 2019; Guo et al., 2024). The UPR is mediated by three evolutionarily conserved sensors: inositol-requiring enzyme 1α (IRE1α); protein kinase R (PKR)-like ER kinase (PERK); and activating transcription factor 6 (ATF6) (Casas-Martinez et al., 2024). Under proteostatic equilibrium, these sensors remain sequestered by the ER chaperone BiP/GRP78. During ERS, misfolded protein overload competitively dissociates BiP/GRP78 from the sensors, enabling their oligomerization and activation. PERK activation catalyzes eIF2α phosphorylation, which transiently attenuates global translation while paradoxically enabling ATF4-mediated transcriptional upregulation of pro-apoptotic factor CHOP. ATF4 and CHOP synergistically stimulate the expression of genes related to apoptosis, autophagy, and antioxidant responses (Lu et al., 2024). IRE1α endoribonuclease activity executes XBP1 mRNA splicing, generating sXBP1 for UPR gene activation, while concomitantly activating apoptosis signal-regulating kinase 1 (ASK1)-JNK1 signaling (Brozzi et al., 2015; Lu et al., 2024). Activated ATF6 translocates to the Golgi for proteolytic cleavage, yielding a transcriptionally active fragment that represses anti-apoptotic Bcl-2 proteins while inducing pro-apoptotic effectors. In myocytes, ATF6 hyperactivation provokes caspase-9-mediated intrinsic apoptosis via mitochondrial permeabilization (Kapuy, 2024).

Akt (protein kinase B, PKB), a pivotal node in insulin receptor substrate (IRS) signaling, coordinates cellular proliferation, survival, and metabolic homeostasis through phosphorylation-dependent regulation of downstream effectors (Viglietto et al., 2002; Bao et al., 2004; Li et al., 2007). Following insulin receptor activation, phosphatidylinositol-3,4,5-trisphosphate (PIP3) recruits Akt to the plasma membrane via pleckstrin homology (PH) domain interaction, enabling PDK1-mediated phosphorylation at Thr308 within the activation loop (Stephens et al., 1998). Concomitant mTOR complex 2 (mTORC2)-dependent phosphorylation at Ser473 in the hydrophobic motif confers full catalytic competence (Zeng et al., 2007). Activated Akt modulates downstream targets, including FoxOs, cell cycle regulators, mTOR, and GSK3, to regulate diverse physiological functions (Manning and Cantley, 2007; Vantler et al., 2023).

ERS-induced apoptosis may play a pivotal role in diabetic skeletal muscle atrophy. However, the specific mechanisms by which ERS regulates skeletal muscle atrophy in diabetes remain unclear. This research aims to explore the molecular mechanisms of ERS-mediated skeletal muscle atrophy in diabetic mice. By comparing skeletal muscle alterations, ERS marker expression, and apoptosis between diabetic and control groups, we seek to provide new insights into the pathogenesis of diabetic muscle atrophy and theoretical support for developing ERS-targeted therapies.

## 2 Materials and methods

### 2.1 Data collection and processing

The GSE22309 dataset was retrieved from the GEO database (https://www.ncbi.nlm.nih.gov/geo/) using the keyword “diabetes.” This dataset included human skeletal muscle samples from insulin-sensitive individuals and insulin-naïve diabetic patients. Differentially expressed genes (DEGs) were identified using GEO2R (|logFC| ≥ 1, FDR < 0.05). Gene Ontology (GO) analysis was conducted with DAVID and visualized with Cytoscape.

### 2.2 Animal experiments

All animal experiments were approved by the Animal Experimental Ethics Committee (IACUC-2303040). Leptin receptor-deficient *Db/db* mice (n = 7, 8-week-old, male) were employed as a type 2 diabetes model, while age-matched male C57BL/6J mice (n = 7) served as normal controls (Pandey and Dvorakova, 2020). Following 16 weeks of standardized housing under specific pathogen-free conditions, skeletal muscle tissues were collected for molecular characterization. All experimental animals were generously provided by Harbin Medical University.

### 2.3 Transcriptome data analysis

RNA from the hindlimb skeletal muscles of 5 control and 4 *Db/db* mice was sequenced on the Illumina platform by Metware Biotechnology Co., Ltd. (Wuhan, China).

### 2.4 Body weight and random blood glucose monitorin

Body weight was measured using an electronic balance (0.1 g resolution). Mice were briefly transferred to an empty cage for consistent weighing. Random blood glucose was assessed without fasting: a needle lightly punctured the tail vein, the first blood droplet was discarded, and subsequent droplets were analyzed via glucometer.

### 2.5 Intraperitoneal glucose tolerance test

Following 12-h fast. A sterile needle was used to puncture the tail tip, and blood droplets were collected for baseline (0-min) glucose measurement. Then glucose (2 g/kg) was administered i. p. Blood glucose was re-measured at 30, 60, and 120 min post-injection (same method) (Zhang Y. et al., 2023).

### 2.6 Antibodies

Anti-SOD (1:1,000), anti-p-eIF2α (1:1,000), anti-GAPDH (1:10,000), anti-ATF6 (1:1,000), anti-eIF2α (1:1,000), anti-p-JNK (1:1,000), anti-JNK (1:1,000), anti-CYTC (1:1,000), anti-Atrogin1 (1:1,000), anti-BiP (1:1,000), anti-Bax (1:1,000), anti-Caspase3 (1:1,000), anti-MYH4 (1:1,000), anti-CHOP (1:1,000), anti-MYOM1 (1:1,000) antibodies were purchased from Proteintech. Anti-TNNI2 (1:1,000), anti-CAT (1:1,000) antibodies were obtained from Affinity Biosciences (Qinke Biotechnology Co., Ltd.). Anti-Bcl2 (1:500) antibody was purchased from Wanlei Biotechnology Co., Ltd.

### 2.7 Immunoblotting analysis

Hind limb skeletal muscle tissues of mice were homogenized in pre-chilled RIPA buffer and centrifuged at low speed (3,000 rpm, 10 min, 4°C). The supernatant was collected and vortexed intermittently (20 s every 5 min for 30 min total). After centrifugation at 12,000 rpm for 30 min (4°C), the clarified supernatant was collected. Protein concentration was determined by BCA assay, and samples were adjusted to load 80 μg protein per well. The samples were mixed with 5× loading buffer at a 1:4 ratio, boiled at 100°C for 8 min, and separated by SDS-PAGE. Proteins were transferred to PVDF membranes (0.45 μm pore size) via wet transfer. Membranes were blocked for 90 min at room temperature, incubated with primary antibodies (4°C, 16 h) and HRP-conjugated secondary antibodies (25°C, 1 h). Protein bands were visualized by ECL and quantified using ImageJ software for normalization and statistical analysis.

### 2.8 Laminin staining

Immunofluorescence staining of hindlimb skeletal muscle (5 μm cryosections) was performed as follows: Tissues were fixed in 4% PFA, embedded in OCT compound, and sectioned using a cryostat. Antigen retrieval was performed by heating sections in citrate buffer (95°C, 20 min). After PBS washes, sections were blocked with blocking buffer (25°C, 30 min) and incubated with anti-laminin antibody (4°C, 16 h). Secondary antibody labeling was conducted under light-protected conditions (25°C, 1 h). Nuclei were counterstained with DAPI (5 min), and slides were mounted with antifade mounting medium. Images were acquired using confocal microscopy.

### 2.9 Statistical analysis

Data were analyzed using GraphPad Prism and expressed as mean ± SEM. Group comparisons were performed using Student’s t-test. The normality of data for all parametric tests (Student’s t-test) was verified using the Shapiro-Wilk test. Significance was set at *P < 0.05 and **P < 0.01.

## 3 Results

### 3.1 Differential gene expression in diabetic skeletal muscle

**FIGURE 1:**
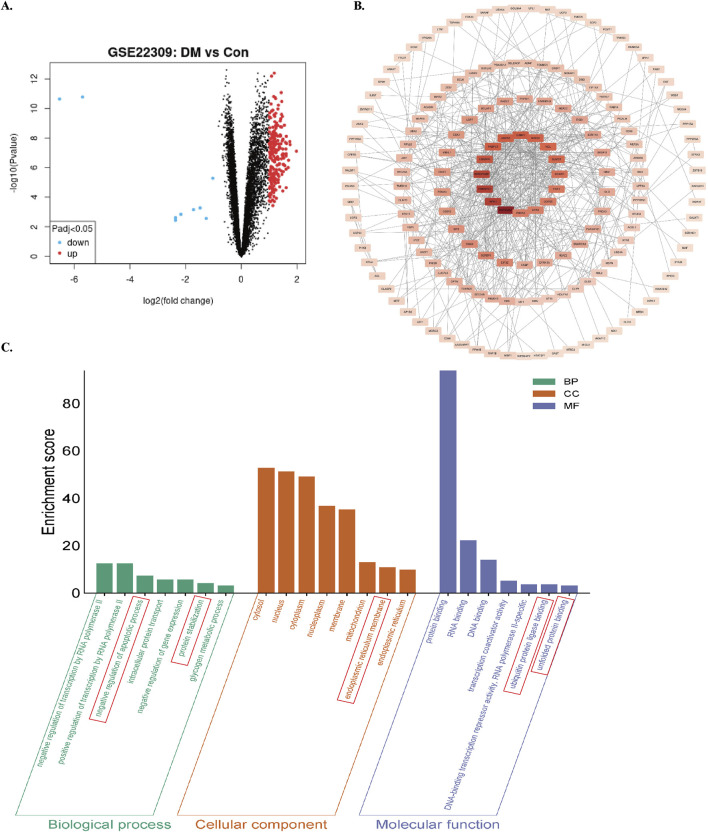
Screening and enrichment analysis of DEGs in the GSE22309 dataset. **(A)** Volcano plot of DEGs comparing skeletal muscle from insulin-sensitive individuals (Con) and untreated diabetic patients (DM). using red/blue color dots to denote upregulation and downregulation. **(B)** PPI network of DEGs. **(C)** GO enrichment analysis of DEGs. Enrichment score reflects the number of enriched genes; higher values indicate stronger enrichment.

Differentially expressed genes (DEGs) were screened using GEO2R-normalized microarray data from the GEO database (GSE22309 dataset) with thresholds of |log2Fold Change| ≥1 and FDR < 0.05. A total of 227 diabetes mellitus (DM)-related DEGs were identified (Figure 1A). Protein-protein interaction network analysis conducted through the STRING database revealed gene clusters associated with ER homeostasis and apoptosis (Figure 1B). Gene Ontology (GO) enrichment analysis demonstrated significant enrichment in biological processes (BPs) including apoptosis regulation, proteostasis maintenance, and glycogen metabolism. Cellular components (CCs) were predominantly localized to the nucleus, cytoplasm, mitochondria, and ER. Molecular functions (MFs) involved protein binding, nucleic acid binding, ubiquitin-protein ligase interactions, and unfolded protein binding (Figure 1C). These bioinformatic analyses collectively indicate dysregulation of ER stress modulation, apoptotic damage, and proteostatic control in skeletal muscle of diabetic patients.

### 3.2 Upregulated ER stress and apoptosis-associated genes in diabetic murine skeletal muscle

**FIGURE 2:**
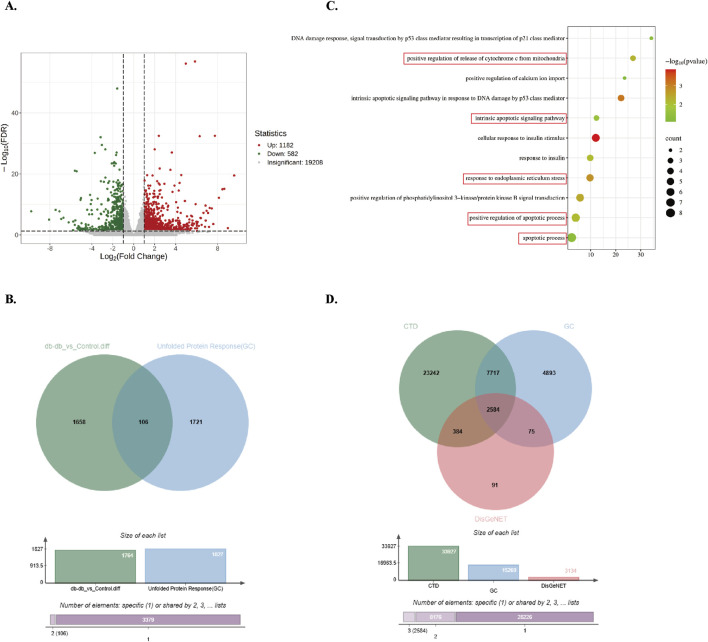
Screening and enrichment analysis of *Db/db*-associated DEGs. **(A)** Volcano plot of *Db/db*-related DEGs, using red/blue color scales to denote upregulation and downregulation. **(B)** Venn diagram showing integration between DEGs and UPR-related genes from GeneCards. **(C)** GO BP enrichment analysis of overlapping genes. Dot size reflects gene count; color intensity represents P-value (darker = lower P). **(D)** Venn diagram of T2DM-related genes from DisGeNET, GeneCards, and CTD.

To further explore differential gene expression patterns, transcriptome sequencing was conducted on hindlimb skeletal muscle tissues from control and *Db/db* mice. DEGs were filtered using thresholds of |log2Fold Change| ≥ 1 and FDR <0.05, resulting in 1,764 DEGs (1,182 upregulated and 582 downregulated in *Db/db* mice) (Figure 2A). Cross-referencing analysis with 1,827 UPR-associated genes from the GeneCards database identified 106 overlapping genes (Figure 2B). Gene Ontology (GO) enrichment analysis demonstrated significant enrichment of BPs related to ER stress, insulin response, and apoptosis (Figure 2C). Furthermore, intersection analysis of 2,584 type 2 diabetes-associated genes curated from CTD, GeneCards, and DisGeNET databases revealed substantial overlap with ER stress and apoptosis-related genes (Figure 2D). Upregulated ER Stress and Apoptosis-Associated Genes in Diabetic Murine Skeletal Muscle: Integrated multi-omics analyses reveal a significant association between ER stress activation and apoptotic pathway enrichment in diabetic murine models.

### 3.3 Metabolic characterization of diabetic murine models

**FIGURE 3:**
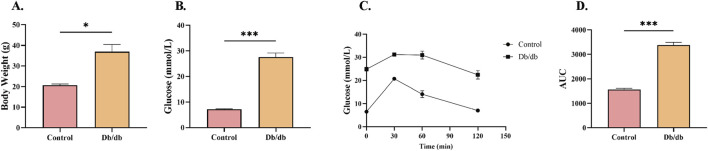
Metabolic Profile Characterization in Control and *Db/db* Murine models. **(A)** Body weight comparison at 8 weeks post-modeling. **(B)** Random blood glucose measurements between Control and *Db/db* cohorts. **(C)** Intraperitoneal glucose tolerance test (IPGTT) trajectories. **(D)** Quantitative area under the curve (AUC) analysis of glycemic responses. Data: mean ± SEM. *P < 0.05 was considered statistically significant. n = 3.

To validate the establishment of T2DM in murine models, bodyweight, random blood glucose levels, and glucose tolerance were systematically assessed in 8-week-old mice. *Db/db* mice demonstrated significantly greater bodyweight relative to controls (Figure 3A). The random blood glucose concentrations in diabetic mice were substantially elevated compared to controls (Figure 3B). Intraperitoneal glucose tolerance testing (IPGTT) further revealed impaired glycemic regulation in *Db/db* mice pared to control mice (Figure 3C). Quantification of the glucose tolerance curve area under the curve (AUC) showed significantly elevated values in diabetic versus controls (Figure 3D). These findings collectively demonstrate disrupted glucose homeostasis in *Db/db* mice, confirming successful T2DM model establishment.

### 3.4 ERS activation in *Db/db* murine models

**FIGURE 4:**
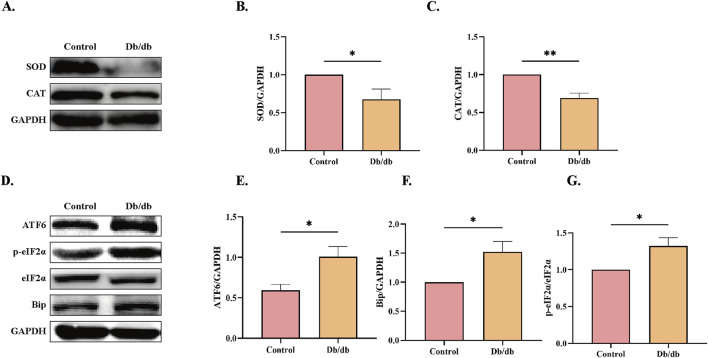
ERS Protein Levels in Control vs. *Db/db* Mice. **(A)** superoxide dismutase (SOD) and catalase (CAT) protein levels. Quantitative analysis of **(B)** SOD, n = 5, **(C)** CAT, n = 4. **(D)** Activating transcription factor 6 (ATF6), immunoglobulin heavy chain binding protein (Bip), and Phosphorylation of eukaryotic initiation factor-2α (p-eIF2α) protein levels. Quantitative analysis of **(E)** ATF6, n = 4, **(F)** Bip, n = 4, **(G)** p-eIF2α, n = 3. Data: mean ± SEM. *P < 0.05 was considered statistically significant.

ERS has been mechanistically linked to oxidative stress, ubiquitin-proteasome pathway activation, and inflammatory signaling (Zhang H. et al., 2023). The quantity levels of critical antioxidant enzymes superoxide dismutase (SOD) and catalase (CAT), which counteract oxidative damage (Jomova et al., 2024), were significantly downregulated in *Db/db* mice compared to Control cohorts (Figures 4A–C), indicative of exacerbated oxidative stress. Furthermore, proteomic analysis revealed elevated expression of ERS markers including the ER transmembrane sensor ATF6, phosphorylated eukaryotic initiation factor 2α (p-eIF2α) as a downstream target of PERK signaling, and the ER chaperone BiP/GRP78 in *Db/db* mice compared to control mice (Figures 4D–G). These findings collectively demonstrate concomitant augmentation of oxidative stress and ERS pathways in diabetic murine models.

### 3.5 Enhanced apoptotic activation in *Db/db* murine models

**FIGURE 5:**
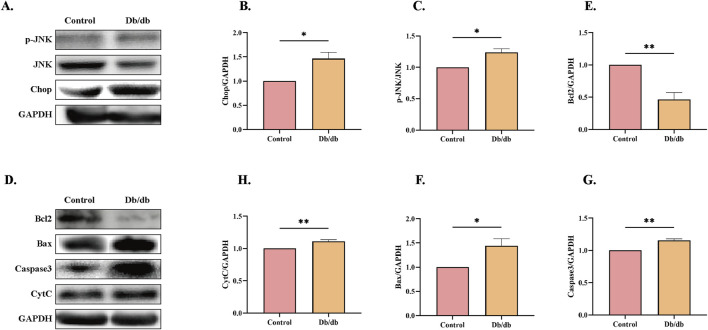
Apoptotic Pathway Activation Profile in Control and *Db/db* Murine models. **(A)** Immunoblot analysis of phosphorylated c-Jun N-terminal kinase (p-JNK) and C/EBP Homologous Protein (CHOP). Quantitative analysis of **(B)** p-JNK, n = 3, **(C)** CHOP, n = 3. **(D)** Protein levels of Cleaved caspase-3 (c-Casp3), Cytochrome c (Cyt c), B-cell lymphoma 2 (Bcl-2), and Bcl-2-associated X protein (Bax). Quantitative analysis of **(E)** Bcl-2, n = 3, **(F)** Bax, n = 3, **(G)** c-Casp3, n = 3, **(H)** Cyt c, n = 4. Data: mean ± SEM. *P < 0.05 was considered statistically significant.

To further investigate whether ERS induces elevated apoptosis in *Db/db* mice, we performed protein-level analyses. CHOP and JNK, downstream targets of the PERK and IRE1α pathways, are well-known apoptosis-related proteins (Lakshmanan et al., 2011). CHOP-deficient mice exhibit reduced caspase-3 activation, increased Bcl-2/Bax ratios, and attenuated apoptosis in cardiac tissues (Fu et al., 2010). Additionally, colistin-induced JNK activation has been shown to significantly elevate the Bax/Bcl-2 ratio (Lu et al., 2017). Our results revealed increased expression of CHOP and phosphorylated JNK (p-JNK) in *Db/db* mice compared to control mice (Figures 5A–C). Furthermore, we assessed the levels of apoptosis-related protein markers and observed elevated expression of pro-apoptotic proteins, including cleaved caspase-3 (C-Caspase3), cytochrome C (CytC), and Bax, alongside reduced levels of the anti-apoptotic protein Bcl-2 in *Db/db* mice versus control mice (Figures 5D–H). These findings collectively indicate that ERS contributes to enhanced apoptosis in *Db/db* mice.

### 3.6 Reduced Akt activity and activation of E3 ubiquitin ligase in *Db/db* murine models

**FIGURE 6:**
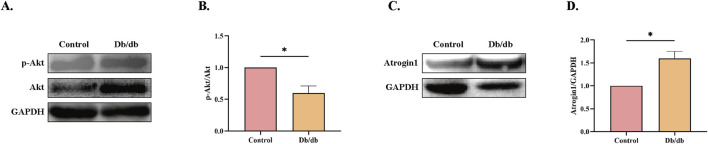
Protein Levels in Control vs. *Db/db* Mice. **(A)** Phosphorylated Protein Kinase B (p-Akt) protein levels. **(B)** Quantification of p-Akt. **(C)** Muscle Atrophy F-box protein 1 (Atrogin-1) protein levels. **(D)** Quantification of Atrogin-1. Statistical significance as above. Data: mean ± SEM. *P < 0.05 was considered statistically significant. n = 3.

A significant decrease in phosphorylated Akt (p-Akt) levels was observed in *Db/db* mice compared to control mice (Figures 6A,B). Additionally, Atrogin-1 protein levels were markedly elevated compared to the Control (Figures 6C,D). These results indicate that ER stress in *Db/db* mice likely reduces Akt activity, leading to the activation of the E3 ligase Atrogin-1 and subsequent skeletal muscle atrophy.

### 3.7 Skeletal muscle atrophy in *Db/db* murine mice

**FIGURE 7:**
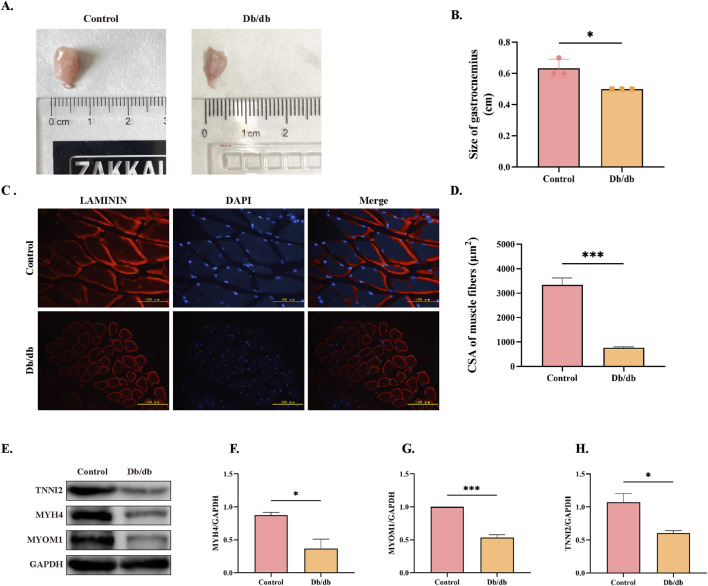
Morphological and Structural Protein Expression Differences in Skeletal Muscle Between Control and *Db/db* Murine Mice. **(A)** Gross anatomy of gastrocnemius muscles. **(B)** Quantification of gastrocnemius muscle mass. **(C)** Laminin immunofluorescence staining of skeletal muscle cross-sections, Scale bar = 100 μm. **(D)** Quantification of muscle fiber cross-sectional area (CSA). **(E)** Western blot analysis of skeletal muscle structural proteins: Troponin I, Fast Skeletal Muscle9 (TNNI2), Myosin Heavy Chain 4 (MYH4), and Myomesin 1 (MYOM1). Quantitative analysis of **(F)** MYH4, **(G)** MYOM1, **(H)** TNNI2. Data: mean ± SEM. *P < 0.05 was considered statistically significant. n = 3.

To assess skeletal muscle atrophy in *Db/db* mice, we first examined gastrocnemius muscle morphology, which revealed a significant reduction in muscle mass compared to control mice (Figures 7A,B). Immunofluorescence staining of laminin further confirmed atrophy, demonstrating a marked decrease in the cross-sectional area (CSA) of muscle fibers in *Db/db* mice compared to control mice (Figures 7C,D). Consistent with these morphological changes, Western blot analysis showed significantly reduced expression levels of key skeletal muscle structural proteins—TNNI2, MYH4, and MYOM1—in *Db/db* mice compared to controls (Figures 7E–H). Together, these findings provide conclusive evidence that *Db/db* mouse exhibit skeletal muscle atrophy.

## 4 Discussion

Maintaining redox homeostasis is crucial for normal physiological functions. Under pathological conditions, oxidative stress disrupts this balance and participates in the pathogenesis of multiple diseases including DM, inflammatory responses, and tumors (Martemucci et al., 2023). As a vital component of cellular stress responses, ERS interacts closely with oxidative stress, the UPS, and inflammatory signaling pathways through molecular mechanisms (Liu et al., 2023). SOD and CAT, serving as the first line of defense against oxidative stress, constitute the most robust protective barrier against oxidative damage (Jomova et al., 2024). Glutathione (GSH), a central antioxidant, maintains redox homeostasis in biological systems. Studies in the epididymis of T2DM rodents demonstrated significantly reduced glutathione peroxidase (GPx) activity and elevated glutathione reductase (GR) levels (Diniz et al., 2022). Another study further revealed that diminished GSH pools in diabetic systems exacerbate oxidative stress and inflammatory responses through redox imbalance (Dawi et al., 2024). These findings collectively imply that disrupted glutathione metabolism may underlie diabetes-associated tissue dysfunction. Research have revealed that oxidative stress disrupts nascent ER protein folding, triggering the UPR to handle misfolded proteins and activating downstream stress sensors that induce ER stress (Peserico et al., 2020). Our results demonstrated that diabetic mice exhibited elevated oxidative stress levels accompanied by reduced protein expression of antioxidant enzymes SOD and CAT. Concurrently, increased protein levels of ER chaperone Bip, UPR stress sensors ATF6 and IRE1α, along with their downstream regulators p-eIF2α, CHOP, and p-JNK were observed. Persistent ER stress ultimately leads to apoptosis-mediated cell death (Yang et al., 2008), while experimental evidence shows that knocking down CHOP or JNK protein levels attenuates apoptosis induction (Yang et al., 2021). The release of CytC into cytoplasm represents a critical event in the mitochondrial-dependent intrinsic apoptotic pathway, where cytosolic CytC combines with apoptotic protease-activating factors to initiate caspase cascade reactions, ultimately causing cellular damage (Wang et al., 2016). Our results showed upregulation of cleaved Caspase-3, Bax and CytC (pro-apoptotic) with concomitant downregulation of Bcl-2 (anti-apoptotic), indicating the occurrence of apoptosis that directly contributes to skeletal muscle atrophy.

Skeletal muscle atrophy primarily arises from three key mechanisms: increased proteolysis, reduced protein synthesis, and impaired myofiber regeneration. These processes are regulated by multiple molecular pathways, including the ubiquitin-proteasome system (UPS), autophagy-lysosomal pathway (ALP), calpain system, caspase pathway, IGF-1/Akt protein synthesis pathway, myostatin signaling, and muscle satellite cells (Ji et al., 2022). Among these, the PI3K/Akt/mTOR signaling pathway plays a central role in modulating protein synthesis in skeletal muscle. Activation of Akt inhibits FoxO-mediated transcription of muscle-specific E3 ubiquitin ligases, such as MuRF1 and Atrogin-1, while simultaneously promoting protein synthesis (Egerman and Glass, 2014). Chronic ER stress has been shown to impair the RTK/PI3K/AKT signaling pathway (Qin et al., 2010; Lu et al., 2017). For example, ER stress in aged rat livers suppresses insulin signaling, inhibits Akt, and upregulates FOXO expression (Kim et al., 2019). FoxOs are transcription factors that regulate E3 ubiquitin ligases. When inhibiting the Akt pathway, nuclear FoxOs promote the expression of MuRF1/atrogin-1, activating the UPS and driving protein degradation and skeletal muscle atrophy (Ji et al., 2022). Similarly, dietary selenium deficiency or excess in rainbow trout modulates the Akt/FoxO3a pathway, accelerating ubiquitin-mediated muscle protein degradation (Zhang et al., 2022).

In our research, we observed reduced Akt activity and elevated Atrogin-1 expression in the skeletal muscle of diabetic mice compared to control mice, accompanied by significant muscle atrophy. This muscular degeneration inevitably leads to impaired motor function, severely impacting the quality of life of affected individuals and imposing substantial personal, familial, and societal burdens.

While this study revealed significant dysregulation of ERS-related molecular markers in diabetic muscle atrophy, the causal relationships remain to be directly validated through functional interventions. Notably, previous investigations have demonstrated that 4-phenylbutyric acid (4-PBA), a chemical chaperone and ERS inhibitor, can ameliorate triptolide (TP)/lipopolysaccharide (LPS)-induced ERS-associated apoptosis and hepatic oxidative stress (Cheng et al., 2024). Furthermore, emerging evidence indicates that pharmacological inhibition of ERS with Salubrinal effectively attenuates ferroptosis and cellular damage in diabetic myocardial ischemia/reperfusion injury (Li et al., 2020). Based on these mechanistic insights, we recommend the following strategies to establish causal relationships: (1) Pharmacological validation using FDA-approved ERS inhibitors (e.g., 4-PBA or tauroursodeoxycholic acid [TUDCA]) in both *Db/db* mice and diabetic myotube models to assess potential reversal of muscle atrophy phenotypes; (2) Genetic validation through targeted knockdown of core ERS regulators (such as PERK, ATF6, or IRE1α) to systematically evaluate their regulatory effects on apoptotic pathways and ubiquitin-proteasome-mediated protein degradation. These investigations will advance the development of ERS-targeted therapies for diabetic sarcopenia.

## 5 Conclusion

**FIGURE 8:**
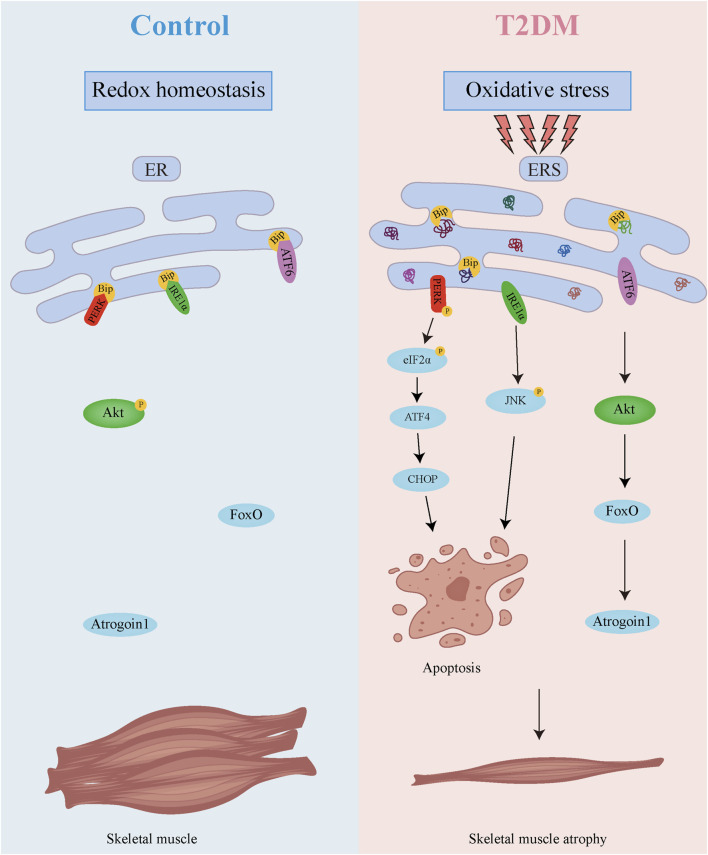
Mechanism diagram of skeletal muscle atrophy in T2DM.

As demonstrated in Figure 8, our research uncovers a dual regulatory mechanism underlying diabetic muscle atrophy. The diabetic skeletal muscle microenvironment exhibits elevated oxidative stress and significantly enhanced ER stress, which promotes direct muscle atrophy through ER stress sensor-mediated apoptosis. Concurrently, sustained ER stress suppresses Akt activity while upregulating the muscle-specific E3 ubiquitin ligase Atrogin-1, thereby accelerating proteolysis and inducing indirect muscle wasting. These findings provide crucial mechanistic insights into diabetic skeletal myopathy, highlighting the ER stress signaling network as a promising therapeutic target for mitigating muscle atrophy in diabetes. Future studies should elucidate the long-term consequences of ER stress modulation, identifying additional molecular players in this pathological cascade, and developing comprehensive intervention strategies to prevent and treat this debilitating complication.

## Data Availability

The raw data supporting the conclusions of this article will be made available by the authors, without undue reservation.
